# Recommendations for additional imaging of abdominal imaging examinations: frequency, benefit, and cost

**DOI:** 10.1007/s00330-019-06382-7

**Published:** 2019-08-26

**Authors:** Sabine A. Heinz, Thomas C. Kwee, Derya Yakar

**Affiliations:** Medical Imaging Center, Department of Radiology, University Medical Center Groningen, University of Groningen, Hanzeplein 1, P.O. Box 30.001, 9700 RB Groningen, the Netherlands

**Keywords:** Abdomen, Costs, Diagnostic imaging, Referral and consultation

## Abstract

**Objectives:**

To investigate the frequency, determinants, clinical implications, and costs of recommendations for additional imaging (RAIs) in secondary interpretations of abdominal imaging examinations.

**Methods:**

This retrospective study included 2225 abdominal imaging examinations from outside institutions that were reinterpreted as part of standard clinical care at a tertiary care center in a one-year time frame.

**Results:**

Two hundred forty-six RAIs were present in 231 of 2225 reports (10.4%) of secondary abdominal imaging interpretations. Patient age and experience of the radiologist who performed the secondary interpretation were independently significantly associated with the presence of an RAI (both *p* = 0.002), with odds ratios of 0.99 per year increase in patient age (95% confidence interval [CI], 0.98–1.00) and 1.06 per year increase in experience of the radiologist (95% CI, 1.02–1.10). If followed, RAIs changed clinical management in 31.2%. Total costs of all 246 RAIs, whether performed or not by the referring physicians, amounted to €71,032.21, thus resulting in €31.92 per secondary abdominal imaging interpretation. Total costs of the 140 RAIs that were actually performed by the referring physicians amounted to €42,683.08, resulting in €19.18 per secondary abdominal imaging interpretation.

**Conclusions:**

The frequency of RAIs in reports of secondary interpretations of abdominal imaging examinations (which appear to be affected by patients’ age and radiologists’ experience) and associated costs are non-negligible. However, RAIs not infrequently change clinical management. The presented data may be helpful to radiology departments and healthcare policy makers to make well-informed decisions on the value and facilitation of the practice of secondary interpretations.

**Key Points:**

*• Frequency of recommendations for additional imaging (RAIs) in secondary interpretations of abdominal imaging examinations at a tertiary care center is approximately 10.4%.*

*• RAIs appear to be more frequently issued in younger patients and by more experienced radiologists, and if followed by referring clinicians, change clinical management in about one third of cases.*

*• RAI costs per secondary interpretation in the Dutch Healthcare system are €31.92 (considering all RAIs) or €19.18 (considering only those RAIs that are actually performed).*

**Electronic supplementary material:**

The online version of this article (10.1007/s00330-019-06382-7) contains supplementary material, which is available to authorized users.

## Introduction

When patients present at or are referred to a tertiary care center, their treating physicians may submit a request to re-evaluate imaging examinations that were recently performed and interpreted at another hospital [1, 2]. Reinterpretations can spare patients unnecessary repeated imaging examinations with associated costs, discomfort, and potential side-effects [3, 4]. In addition, a secondary assessment by tertiary care subspecialized radiologists may improve diagnostic interpretation and can change clinical management, as demonstrated by several previous studies [2, 5–9].

Although secondary interpretations have several potential advantages, they may contain a recommendation for additional imaging (RAI). For radiologists, it is not uncommon to make such recommendations for reasons such as reducing uncertainty, evaluating an indeterminate finding with a more sensitive modality, or assessing the temporal stability of a lesion [10–12]. However, a disadvantage of RAIs is that they impose healthcare costs. In the USA, growth rates for secondary interpretations between 2003 and 2016 have been reported to range from 4.3 to 35.7%, depending on the imaging modality and subspecialty [13]. Review of our own hospital records revealed a 150% increase over the past 5 years, and around 3.6% of all procedures performed at our department currently constitute of secondary interpretations, the vast majority for abdominal imaging examinations. Therefore, total costs for RAIs that are given in secondary interpretations have likely also grown over the years. For secondary interpretations, there is currently a lack of data on the frequency of RAIs, the factors that are associated with the addition of an RAI to the report by the radiologist, their clinical implications, and the costs of these RAIs. These data are important for radiology departments and healthcare policy makers to make well-informed decisions on the value and facilitation of the practice of secondary interpretations.

The purpose of this study was therefore to investigate the frequency, determinants, clinical implications, and costs of recommendations for additional imaging (RAIs) in secondary interpretations of abdominal imaging examinations.

## Materials and methods

### Study design

The local institutional review board of the University Medical Center Groningen approved this retrospective, single-center study, and informed consent was waived (number 2017/433). The University Medical Center Groningen is a tertiary care center that provides primary and specialty care to approximately 2.2 million people in the northeast of the Netherlands. All consecutive 3718 secondary interpretations that were performed as part of standard clinical care at the Department of Radiology between November 25, 2016, and November 24, 2017, were reviewed by a research fellow (S.A.H.). Secondary interpretations were included in this study if they concerned an abdominal imaging examination, either alone or in combination with another body region. A secondary interpretation was excluded from this study if it concerned a re-evaluation of an imaging examination that was previously acquired and interpreted at our own institution, if it involved a nuclear medicine examination, if the secondary report was not available in the picture archiving and communication system (PACS), or if the report did not contain a diagnostic interpretation of the imaging examination.

### Practice of secondary interpretations

At our institution, a formal procedure for requests of secondary interpretations is in place. This is the only way a physician can obtain a secondary interpretation by a radiologist. The treating physicians directly contact a subspecialty radiologist to ask permission for a secondary interpretation of an imaging examination performed elsewhere. The radiologist does not review the outside images or report, but interrogates the referring physician for which aim the secondary reading is requested, and then decides if a subspecialty reinterpretation can have added value above the interpretation that was performed elsewhere. After approval by the radiologist, and importing the imaging examination and original report into the PACS, a subspecialty radiologist makes a secondary report (which may or may not include an RAI). The radiologist who performs the reinterpretation is not necessarily the same as the radiologist who granted permission for the secondary reading. Neither the radiologist, nor the department, nor the hospital receives any reimbursement for this secondary interpretation. Furthermore, radiologists and all other medical specialists are paid a fixed salary at our institution, regardless of the number of procedures performed, including RAIs.

### Data extraction

A research fellow (S.A.H.) analyzed all secondary abdominal imaging interpretations, and collected the following variables: patient age, gender, hospital status (inpatient or outpatient) at the time of the secondary interpretation, indication for the imaging examination (infectious, inflammatory, miscellaneous, oncologic, trauma, vascular, miscellaneous), modality and body region of the imaging examination, years of experience of the (most senior) radiologist signing the report of the secondary interpretation (calculated from the completion of residency), and subsequently classifying the radiologist as “less experienced” (≤ 4 years of post-residency experience) or “more experienced” (≥ 5 years of post-residency experience), as well as the presence or absence of an RAI, and whether this RAI resulted in a change in clinical management. Only recommendations specific for further imaging procedures were taken into account. Furthermore, RAIs were excluded if they would have been performed anyway in a specific patient (e.g., additional or follow-up imaging according to a certain guideline or institutional protocol), regardless of the findings on the imaging examination that was submitted for a secondary interpretation. For RAIs without a clearly specified imaging modality, the most appropriate imaging modality was determined by reviewing the report of the secondary imaging interpretation by consensus of two radiologists (T.C.K. and D.Y.).

### Statistical analysis

The frequency of examinations with an RAI as a proportion of the total amount of secondary abdominal imaging interpretations was calculated. Univariate and multivariate logistic regression analyses were performed to determine the association between age, gender, hospital status, indication for the imaging examination, and experience of the (most senior) radiologist who signed the report of the secondary interpretation, with the presence of an RAI. Frequencies of clinical management changes as a result of RAIs were assessed for RAIs that were followed by referring physicians and those that were not, and according to RAIs that were issued by less experienced vs. more experienced radiologists. Costs of all RAIs and costs of RAIs that were actually performed by the treating physicians were calculated according to Dutch Healthcare Authority (Nederlandse Zorgautoriteit, NZa) tariffs (Table 1). *P* values < 0.05 were considered statistically significant. All statistical analyses were performed using IBM Statistical Package for the Social Sciences (SPSS) version 25.

**Table 1:** Overview of imaging costs (€) according to Dutch Healthcare Authority (Nederlandse Zorgautoriteit, NZa) tariffs

Imaging modality	Range of costs (€) per unit examination*	Mean costs (€) per unit examination†
Computed tomography	167.63–172.29	169.96
Endoscopic ultrasonography	508.42	508.42
Endoscopic retrograde cholangiopancreatography	590.19	590.19
Fluoroscopy	134.76	134.76
Mammography	88.75	88.75
Magnetic resonance imaging	213.93–297.95	265.49
FDG-PET/CT	933.79	933.79
Ultrasonography	76.92–84.31	81.85

*Costs differ per unit examination depending on the body region for which the examination is made

†Average of costs for different body regions included in this study

## Results

### Secondary interpretations and patients

**Fig. 1:**
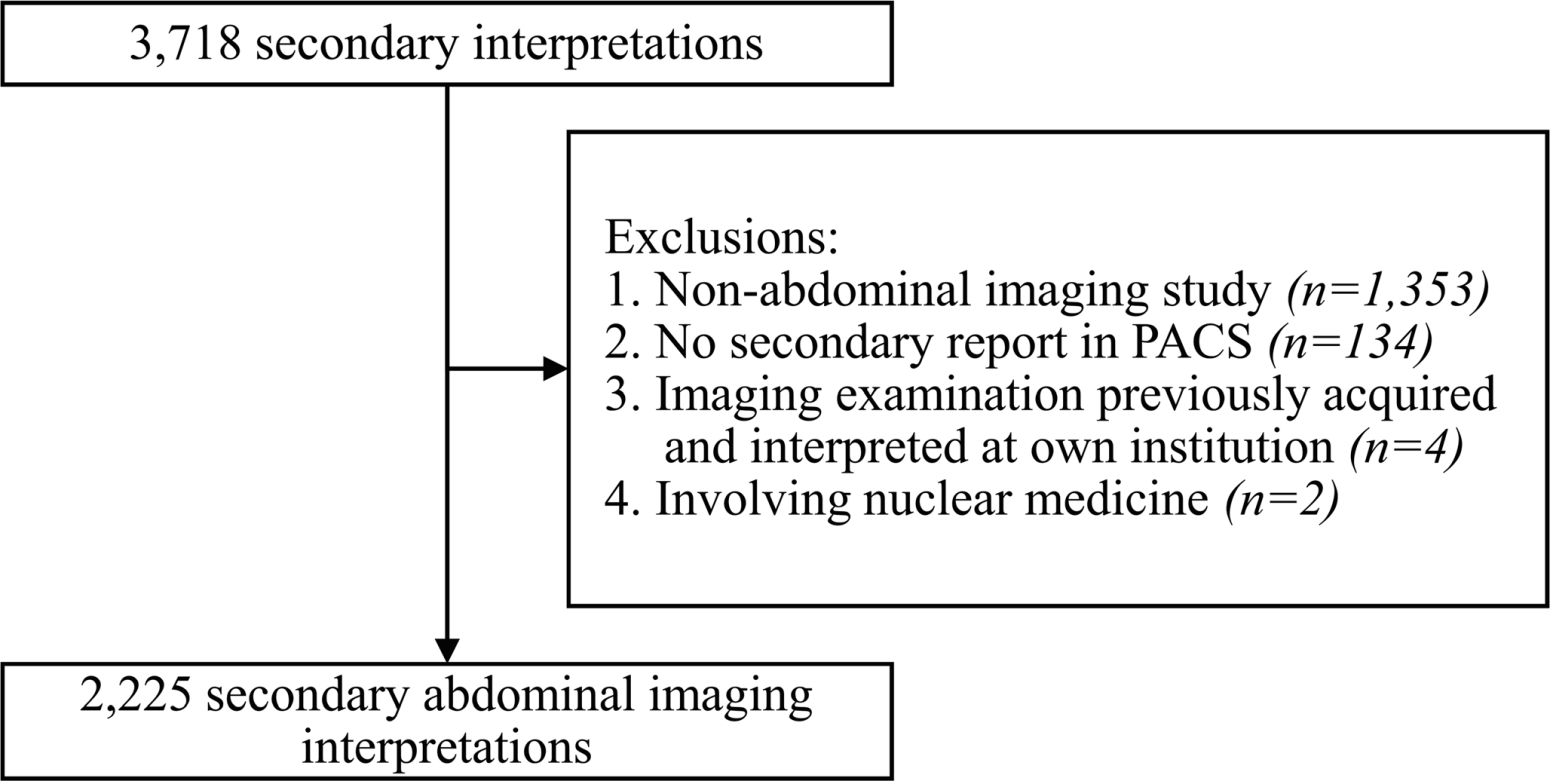
Flowchart showing the number of eligible, excluded and included secondary interpretations

Out of the total number of 3718 examinations with a secondary interpretation, 1353 were excluded as they involved a non-abdominal imaging study, 134 were excluded because there was no secondary report containing a diagnostic interpretation of the imaging examination in the PACS, 4 were excluded because they concerned a re-evaluation of an imaging examination previously acquired and interpreted at our own institution, and 2 were excluded because the secondary interpretation involved a nuclear medicine examination. Thus, 2225 secondary abdominal imaging interpretations remained and were included in this study (Fig. 1). These 2225 imaging examinations were performed in 965 male and 1260 female patients, with a mean age ± SD of 59.6 ± 17.6 years (age range, 0–98 years). The far majority of patients (95.7%) were outpatients, the majority of imaging examinations (76.6%) were performed for oncologic indications, almost all imaging examinations concerned either computed tomography (CT) (71.8%) or magnetic resonance imaging (MRI) (26.1%), the majority of secondary interpretations (85.3%) comprised one imaging examination, and a small majority of imaging examinations (51.0%) exclusively concerned the abdomen. More detailed information on patient and imaging examination characteristics is shown in Table 2. Thirty-three radiologists performed the secondary readings (with one to 542 secondary interpretations per radiologist), and they had a mean experience ± SD of 3.7 ± 3.2 years (range, 0–30 years).

**Table 2:** Patient and abdominal imaging examination characteristics for which a secondary interpretation was requested (*N* = 2225)

Variable	*n* (%)
Sex	
Female	1260 (56.6)
Male	965 (43.4)
Hospital status	
Inpatient	95 (4.3)
Outpatient	2130 (95.7)
Indication for the secondary interpretation	
Infectious	10 (0.4)
Inflammatory	43 (1.9)
Oncologic	1705 (76.6)
Trauma	18 (0.8)
Vascular	21 (0.9)
Miscellaneous	428 (19.2)
Imaging modalities for secondary interpretation*	
CT	1839 (71.8)
Fluoroscopy	14 (0.5)
MRI	668 (26.1)
Ultrasonography	31 (1.2)
X-Ray	10 (0.4)
Number of imaging modalities per secondary interpretation	
1	1898 (85.3)
2	322 (14.5)
3	4 (0.2)
4	1 (0.0)
Body region for secondary interpretation*	
Full abdomen	593 (23.1)
Upper abdomen	454 (17.7)
Lower abdomen	256 (10.0)
Full chest and abdomen	1089 (42.5)
Abdomen in combination with other body regions	129 (5.0)
Scans of other body regions	41 (1.6)
Recommendation for additional imaging (RAI)	
Yes	239 (10.7)
No	1984 (89.2)

*As some secondary imaging interpretations involved an evaluation of multiple imaging modalities, the numbers of imaging modalities and body regions for secondary interpretation are higher than the number of reports included in this study

### RAI frequency

A total of 239 out of 2225 reports contained an RAI. Eight RAIs were excluded because they would have been performed anyway according to institutional protocol. These 8 RAIs all concerned MRI of the uterine cervix in patients with cervical cancer who had only undergone CT at the time of the secondary interpretation. The 231 reports with an RAI that were finally included, corresponded to an RAI frequency of 10.4% (95% confidence interval [CI], 9.2%–11.7%). As 13 reports expressed 2 RAIs and one report expressed 3 RAIs, the total number of RAIs amounted to 246. In 31 of the 246 RAIs (12.6%), the RAI was made due to insufficient quality of the imaging examination, the majority involving MRI (*n* = 23, 74.2%), followed by ultrasonography (*n* = 4, 12.9%), CT (*n* = 3, 9.7%), and mammography (*n* = 1, 3.2%). For MRI, those requests were put forward due to insufficient signal-to-noise ratio/spatial resolution (*n* = 14, 69.1%), patient movement (*n* = 6, 26.1%), and missing sequences (*n* = 3, 13.0%). For ultrasonography, reasons were insufficient quality (*n* = 2, 50%) and a too small number of images provided (*n* = 2, 50%). For CT examinations, those requests were made due to inadequate series presented (*n* = 2, 66.7%) and missing series (*n* = 1, 33.3%). For mammography, the RAI was issued due to low quality (*n* = 1, 100%).

### RAI determinants

Univariate logistic regression analysis showed a significant association of patient age (*p* < 0.001), oncologic indication for the imaging examination (*p* = 0.014), and experience of the radiologist who performed the secondary interpretation (*p* < 0.001), with the presence of an RAI (Table 3). No significant association was found for any of the other variables (Table 3). On multivariate analysis, only patient age and experience of the radiologist who performed the secondary interpretation, remained significantly associated with the presence of an RAI (*p* = 0.002 for both), with odds ratios of 0.99 per year increase in patient age (95% CI, 0.98–1.00) and 1.06 per year increase in experience of the radiologist (95% CI, 1.02–1.10) (Table 4).

**Table 3:** Univariate logistic regression analysis on the association of clinical and radiologic report variables with the presence of an RAI in the report of the secondary interpretation

Variable	Univariate analysis		
	Odds ratio	95% CI	*p* value
Patient age (years, continuous scale)	0.99	0.98–0.99	< 0.001
Patient gender (male vs female)	1.06	0.81–1.38	0.696
Hospital status (in- vs outpatient)	0.84	0.42–1.69	0.623
Indication for the secondary interpretation			
Infectious vs others	2.03	0.43–9.60	0.373
Inflammatory vs others	0.60	0.18–1.96	0.397
Oncologic vs others	0.69	0.52–0.93	0.014
Trauma vs others	1.01	0.23–4.42	0.990
Vascular vs others	1.28	0.38–4.35	0.695
Experience of the radiologist who made the secondary interpretation (years, continuous scale)	1.07	1.03–1.10	< 0.001

*CI* confidence interval

**Table 4:** Multivariate logistic regression analysis on the association of clinical and radiologic report variables with the presence of an RAI in the report of the secondary interpretation

Variable	Multivariate analysis		
	Odds ratio	95% CI	*p* value
Patient age (years, continuous scale)*	0.99	0.98–1.00	0.002
Indication for the secondary interpretation			
Oncologic vs others	0.86	0.61–1.21	0.379
Experience of the radiologist who made the secondary interpretation (years, continuous scale)*	1.06	1.02–1.10	0.002

*CI* confidence interval

*For variables on a continuous scale, the odds ratio indicates the increase or decrease of odds per unit of the scale, i.e., per year

### RAI types

**Fig. 2:**
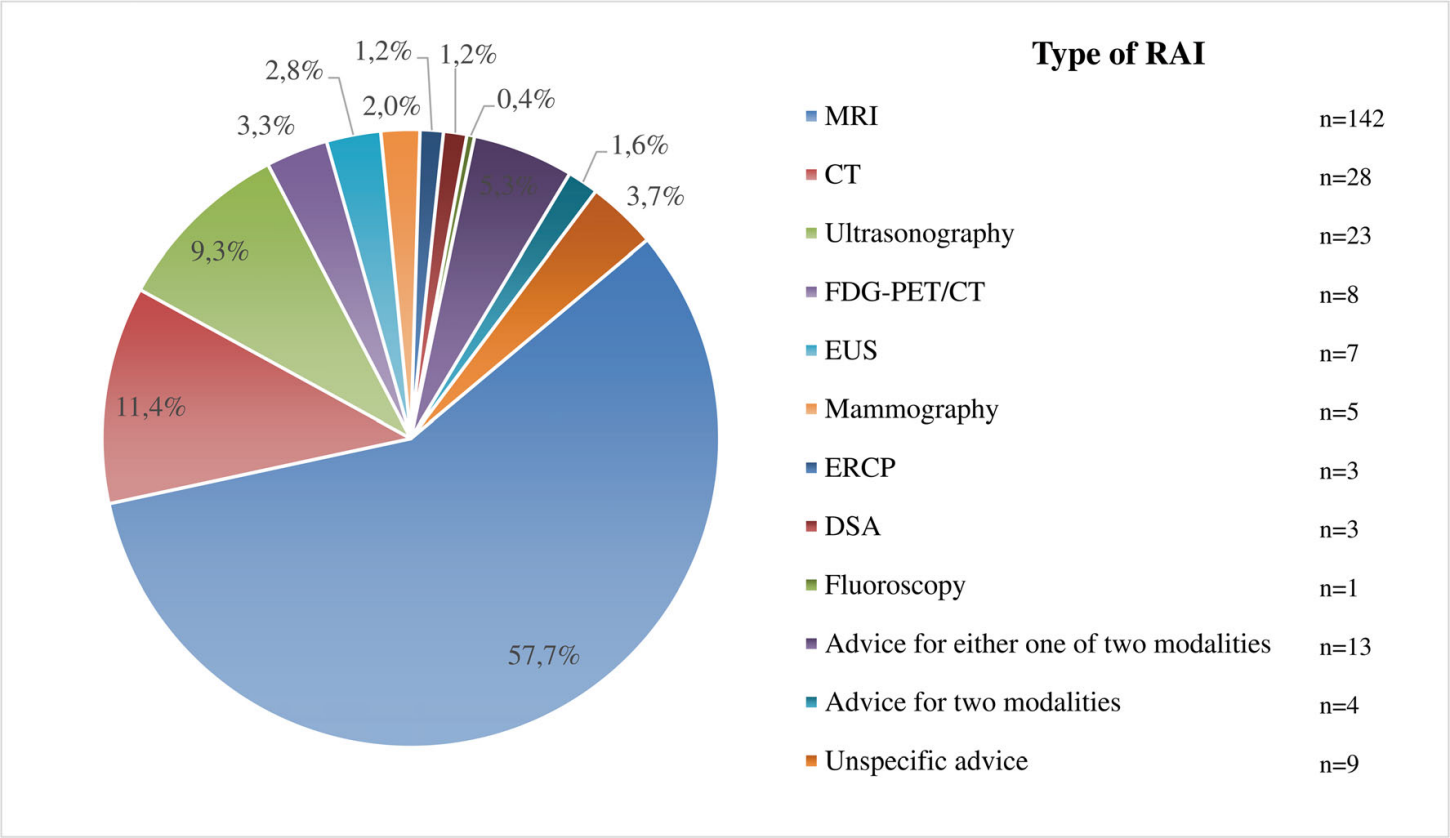
Frequencies of RAI types

The majority of the 246 RAIs involved additional MRI (*n* = 142, 57.7%), followed by CT (*n* = 28, 11.4%) and ultrasonography (*n* = 23, 9.3%). Other requests for additional imaging examinations included ^18^F-fluoro-2-deoxy-D-glucose positron emission tomography/CT (FDG-PET/CT) (*n* = 8, 3.3%), endoscopic ultrasonography (EUS) (*n* = 7, 2.8%), mammography (*n* = 5, 2.0%), digital subtraction angiography (DSA) (*n* = 3, 1.2%), endoscopic retrograde cholangiopancreatography (ERCP) (*n* = 3, 1.2%), and fluoroscopy (*n* = 1, 0.4%). Four RAIs (1.6%) involved an advice for two imaging modalities (mammography and ultrasonography (*n* = 3, 1.2%), MRI and ultrasonography (*n* = 1, 0.4%)). Thirteen RAIs (5.3%) concerned an advice for either one of two modalities (CT or MRI (*n* = 5, 2.0%), MRI or ERCP (*n* = 3, 1.2%), MRI or EUS (n = 3, 1.2%), and MRI or ultrasonography (*n* = 2, 0.8%)), while in 9 RAIs (3.7%), the imaging modality was not specified in the report. Based on a review of the reports of the secondary interpretations, the most appropriate imaging modality for the unspecified RAIs were CT (*n* = 8) and MRI (*n* = 1). All RAI types are summarized in Fig. 2.

### Clinical management changes as a result of RAIs

RAIs that were followed by the referring physicians led to a change in clinical management in 43/138 cases (31.2%), of which the far majority (*n* = 36) were RAIs recommended by less experienced radiologists. RAIs that were not followed would have led to a change in clinical management (if actually followed) in 1/108 cases (0.9%). This recommendation was made by a more experienced radiologist. More detailed information on changes in clinical management is shown in Table 5.

**Table 5:** Changes in clinical management of 246 RAIs, stratified according to reports with RAIs that were followed and reports with RAIs that were not followed by the referring physicians, and according to less experienced radiologists (≤ 4 years of post-residency experience) and more experienced radiologists (≥ 5 years of post-residency experience)

	Change in clinical management					
	Yes	95% CI (%)	No	95% CI (%)	Unknown^*^	95% CI (%)
Reports with RAIs that were followed (*n* = 138)						
Less experienced radiologists	36 (26.1%)	19.3–33.9	35 (25.4%)	18.7–33.1	39 (28.3%)	21.3–36.2
More experienced radiologists	7 (5.1%)	2.3–9.7	12 (8.7%)	4.8–14.3	9 (6.5%)	3.3–11.6
Reports with RAIs that were not followed (*n* = 108)						
Less experienced radiologists	0 (0.0%)	–	7 (6.5%)	2.9–12.3	73 (67.6%)	58.4–75.9
More experienced radiologists	1 (0.9%)	0.1–4.2	2 (1.9%)	0.4–5.8	25 (23.1%)	16.0–31.7

*CI* confidence interval

*It could not be determined if the RAI changed or would have changed clinical management

### RAI costs

Total costs of all 246 RAIs, whether performed or not by the referring physicians, amounted to €71,032.21, thus resulting in €31.92 per secondary abdominal imaging interpretation (Supplemental Table 1). Of these 246 RAIs, 138 (56.1%) were actually followed by the referring physicians. In two cases, both of the suggested possible additional imaging examinations were performed, thus resulting in a total of 140 performed RAIs. Of these 140 performed RAIs, 89 (63.6%) involved MRI, 18 (12.9%) CT, 14 (10%) ultrasonography, 6 (4.3%) EUS, 6 (4.3%) FDG-PET/CT, 4 (2.9%) ERCP, 2 (1.4%) mammography, and 1 (0.7%) DSA. Total costs of these RAIs that were performed by the referring physicians amounted to €42,683.08, resulting in €19.18 per secondary abdominal imaging interpretation (Supplemental Table 2).

## Discussion

The results of this study show that approximately 1 in 10 abdominal imaging examinations that are performed elsewhere and that are presented to a subspecialty radiologist for a secondary interpretation at a tertiary care center are followed by an RAI. Interestingly, RAIs were significantly and independently more frequently issued in younger patients and when radiologists who performed the secondary interpretation had more years of experience. For example, a 16-year-old patient was twice (0.99^69^) as likely to have an RAI in the report than an 85-year-old patient, and a radiologist with 14 years of experience was twice (1.06^12^) as likely to issue an RAI than a radiologist with 2 years of experience. The former may be explained by the fact that younger patients have more disability- or quality-adjusted life years at stake than older patients, and radiologists may have a higher tendency to request further imaging to increase diagnostic certainty in a younger population rather than discarding unclear or indeterminate findings as clinically irrelevant. The latter cannot be completely explained, because it was expected that more experienced radiologists would have gained more expertise and diagnostic confidence, and thus require less RAIs. On the other hand, radiologists with more years of experience may have encountered more cases in which imaging failed to provide a timely and accurate diagnosis, and may therefore have lowered their threshold to issue an RAI to minimize the chance of being involved in malpractice. Nonetheless, it should be noted that the performance of a radiologist remains a complicated topic. Even though many studies have shown the value of experience and subspecialty training [14–17], individual competence cannot always be attributed to experience alone [18, 19]. Therefore, it is difficult to compare performances of radiologists solely based on years of experience or the number of cases read in their career. Personal ability or talent remains difficult to measure. Importantly, of all RAIs that were followed, a considerable proportion of 31.2% led to clinical management changes. This indicates that RAIs issued by subspecialty radiologists in secondary reports may certainly have added value. Interestingly, the far majority of these RAIs that were followed and that led to clinical management changes were issued by less experienced radiologists. This finding is subject to the same considerations as discussed above.

On average, each secondary abdominal imaging interpretation was accompanied by additional RAI costs of €31.92 or €19.18, for all RAIs together and only those RAIs that were actually followed by the referring physicians, respectively. These additional costs can be considered as non-negligible. Moreover, in countries in which the costs for medical imaging are substantially higher (e.g., the USA), the financial burden of these RAIs becomes a relatively larger issue. Furthermore, secondary interpretations require radiologists’ interpretation time. In addition, these imaging examinations are frequently discussed in subsequent multidisciplinary meetings, which also add to the valuable time spent by the radiology team. A comprehensive cost-effectiveness analysis is required to outweigh the benefits of secondary readings against their disadvantages, of which RAIs constitute a non-negligible proportion as shown by the present study. Importantly, the number of secondary interpretations has risen over the past years [13]. If this rise continues, the need to address this type of healthcare overutilization becomes even more imminent. At our institution, there is a formal procedure to submit a request for a secondary interpretation, and the appropriateness of this request is judged by a radiologist. Direct ad hoc requests that circumvent this formal procedure are not granted. We believe this procedure can reduce the number of unnecessary secondary interpretations that do not contribute to patient care. Another interesting issue is the finding that about 1 in 8 RAIs in the present study was due to insufficient quality of the re-evaluated imaging examination. This may be tackled by providing feedback to radiology departments from which these imaging examinations originated. Healthcare systems may also consider enforcing policies to stimulate all tertiary care patients to be referred to and their imaging examinations to be performed in dedicated centers with subspecialty radiologists as timely as possible. This will not only mitigate redundant interpretation time by radiologists working in non-tertiary care centers, but may also reduce the number of subsequent RAIs because of the use of optimized imaging protocols. Furthermore, performing the imaging examination and the evaluation in one place might increase the availability of relevant clinical information expected to be in the electronic patient files. Lastly, and perhaps most importantly, we believe in the importance of registration. Registering the number of requested secondary interpretations according to variables such as disease, requesting specialism, requesting physician, and hospital where the imaging examinations were performed, may help to identify bottlenecks in patient streams, of which secondary interpretation requests are the symptoms. This will provide solid data to radiologists to communicate with other stakeholders (including hospital managements and healthcare insurance companies) to tackle structural problems in patient streams. This may, in turn, also decrease the numbers of secondary interpretations and associated RAIs.

Although there is a relatively large body of literature on the frequency of RAIs in primary interpretations (which have been reported to be around 10.5 to 10.6% in the large-scale studies by Sistrom et al [10] and Mabotuwana et al [20]), there are few studies on this topic for secondary interpretations. Previous studies by Huicochea Castellanos et al [2], Shetty et al [7], and Corrias et al [8] reported RAI frequencies in secondary interpretations ranging between 6% and 19%. However, these studies were limited by small sample sizes, had a focus on hepatopancreatobiliary diseases, and were performed in the USA [2, 7, 8], which may explain the different and widely varying RAI frequencies in these studies. Furthermore, these studies did not investigate determinants and costs of RAIs in secondary interpretations.

This study had some limitations. First, it was performed in a European tertiary care center, where the majority of secondary interpretations are performed for oncologic indications. Furthermore, neither referring physicians nor radiologists have any potential financial incentives to perform secondary interpretations or RAIs at our institution. Therefore, the presented results may not be applicable to other countries and institutions with different patients, and different socioeconomic and medicolegal scenarios. Second, the secondary interpretations in this study consisted of an evaluation of different imaging modalities. Nevertheless, although this may have added heterogeneity to the results, it represents clinical practice.

In conclusion, the frequency of RAIs in reports of secondary interpretations of abdominal imaging examinations (which appear to be affected by patients’ age and radiologists’ experience) and associated costs are non-negligible. However, RAIs not infrequently change clinical management. The presented data may be helpful to radiology departments and healthcare policy makers to make well-informed decisions on the value and facilitation of the practice of secondary interpretations.

## Electronic supplementary material

ESM 1 (DOCX 25 kb)

## Abbreviations

CI: Confidence interval
CT: Computed tomography
DSA: Digital subtraction angiography
ERCP: Endoscopic retrograde cholangiopancreatography
EUS: Endoscopic ultrasonography
FDG-PET: ^18^F-fluoro-2-deoxy-D-glucose positron emission tomography
MRI: Magnetic resonance imaging
NZa: Nederlandse Zorgautoriteit
PACS: Picture archiving and communication system
RAI: Recommendation for additional imaging
SPSS: Statistical Package for the Social Sciences
