# Supplementary material for: Recommendations for additional imaging of abdominal imaging examinations: frequency, benefit, and cost
Source: Eur Radiol. 2019 Aug 26;30(2):1137–44. doi: 10.1007/s00330-019-06382-7 (PMC6957539; doi:10.1007/s00330-019-06382-7)
Supplement: Supplementary file 1 — (DOCX 25 kb) [file 330_2019_6382_MOESM1_ESM.docx]

**Supplemental data**

| **Supplemental Table 1**. RAI type (imaging modality and body region), number of RAIs, costs per unit examination, and total costs of RAIs in this study of 2,225 abdominal secondary interpretations, regardless of whether the RAI was performed or not. | | | | |
| --- | --- | --- | --- | --- |
| RAI type | | No. of RAIs | Costs (€) per unit examination^1^ | Total costs (€) |
| Imaging modality | Body Region |  |  |  |
| MRI^2^ | Liver | 98 | 297.95 | 29,199.10 |
|  | Abdomen | 21 | 297.95 | 6,256.95 |
|  | Prostate | 12 | 297.95 | 3,575.40 |
|  | HPB³ | 4 | 297.95 | 1,191.80 |
|  | Pelvis | 3 | 266.64 | 799.92 |
|  | Brain | 1 | 242.78 | 242.78 |
|  | Breast | 1 | 263.32 | 263.32 |
|  | Lumbar spine | 1 | 213.93 | 213.93 |
|  | Shoulder | 1 | 243.38 | 243.38 |
| CT^4^ | Abdomen | 21 | 167.63 | 3,520.23 |
|  | Chest | 6 | 172.29 | 1,033.74 |
|  | Chest and abdomen | 1 | 339.92 | 339.92 |
| Ultrasonography | Abdomen | 21 | 84.31 | 1,770.51 |
|  | Testis | 1 | 76.92 | 76.92 |
|  | Transvaginal | 1 | 84.31 | 84.31 |
| Unspecified | Chest | 5 | 172.29^5^ | 861.45 |
|  | Abdomen | 3 | 167.63 | 502.89 |
|  | Liver | 1 | 297.95 | 297.95 |
| FDG-PET/CT^6^ | Whole body | 8 | 933.79 | 7,470.32 |
| EUS^7^ | Upper abdomen | 7 | 508.42 | 3,558.94 |
| Mammography | Breast | 5 | 88.75 | 443.75 |
| CT or MRI | Liver | 4 | 232.79^8^ | 931.16 |
|  | Abdomen | 1 | 232.79^9^ | 232.79 |
| DSA^10^ | Portal veins | 3 | 732.64 | 2,197.92 |
| ERCP^11^ | HPB^3^ | 3 | 590.19 | 1,770.57 |
| Mammography and Ultrasonography | Breast | 3 | 170.40 | 511.20 |
| MRI or ERCP | HPB^3^ | 3 | 444.07^12^ | 1,332.21 |
| MRI or EUS | HPB^3^ | 3 | 403.19^13^ | 1,209.57 |
| MRI or Ultrasonography | Liver | 2 | 191.13^14^ | 382.26 |
|  |  |  |  |  |
| MRI and Ultrasonography | Abdomen | 1 | 382.26^15^ | 382.26 |
| Fluoroscopy | Upper gastrointestinal tract | 1 | 134.76 | 134.76 |
| Total RAI costs for all 2,225 secondary abdominal imaging interpretations (€) | | | | 71,032.21 |
| Average RAI costs per secondary abdominal imaging interpretation (€) | | | | 31.92 |
|  | | | | |

Notes:

^1^ according to Dutch Healthcare Authority (Nederlandse Zorgautoriteit, NZa) tariffs

^2^ MRI: magnetic resonance imaging

^3^ HPB: hepato-pancreato-biliary system

^4^ CT: computed tomography

^5^ costs were calculated exploiting the modality of the scan the secondary interpretation was made on

^6^ FDG-PET/CT: ^18^F-fluoro-2-deoxy-D-glucose-positron emission tomography/computed tomography

^7^ EUS: endoscopic ultrasonography

^8^ average of costs for CT and MRI liver

^9^ average of costs for CT and MRI abdomen

^10^ DSA: digital subtraction angiography

^11^ ERCP: endoscopic retrograde cholangiopancreatography

^12^ average costs for MRI HPB and ERCP

^13^ average costs for MRI abdomen and EUS upper abdomen

^14^ average costs for MRI liver and Ultrasonography abdomen

^15^ average costs for MRI and Ultrasonography abdomen

| **Supplemental Table 2**. RAI type (imaging modality and body region), number of RAIs, costs per unit examination, and total costs of RAIs in this study of 2,225 abdominal secondary interpretations for all RAIs that were actually performed. | | | | |
| --- | --- | --- | --- | --- |
| RAI type | | No. of RAIs | Costs (€) per unit examination^1^ | Total costs (€) |
| Imaging modality | Body Region |  |  |  |
| MRI^2^ | Liver | 63 | 297.95 | 18,770.85 |
|  | Abdomen | 14 | 297.95 | 4,171.30 |
|  | Prostate | 10 | 297.95 | 2,979.50 |
|  | Brain | 1 | 242.78 | 242.78 |
|  | Lumbar spine | 1 | 213.93 | 213.93 |
| CT^3^ | Abdomen | 12 | 167.63 | 2,011.56 |
|  | Chest | 5 | 172.29 | 861.45 |
|  | Chest and abdomen | 1 | 339.92 | 339.92 |
| Ultrasonography | Abdomen | 11 | 84.31 | 927.41 |
|  | Breast | 2 | 81.65 | 163.30 |
|  | Testis | 1 | 76.92 | 76.92 |
| EUS^4^ | Upper abdomen | 6 | 508.42 | 3,050.52 |
| FDG-PET/CT^5^ | Whole body | 6 | 933.79 | 5,602.74 |
| ERCP^6^ | HPB^7^ | 4 | 590.19 | 2,360.79 |
| Mammography | Breast | 2 | 88.75 | 177.50 |
| DSA^8^ | Abdomen | 1 | 732.64 | 732.64 |
| Total RAI costs for all 2,225 secondary abdominal imaging interpretations (€) | | | | 42,683.08 |
| Average RAI costs per abdominal secondary abdominal imaging interpretation (€) | | | | 19.18 |
| Notes:  ^1^ according to Dutch Healthcare Authority (Nederlandse Zorgautoriteit, NZa) tariffs  ^2^ MRI: magnetic resonance imaging  ^3^ CT: computed tomography  ^4^ EUS: endoscopic ultrasonography  ^5^ FDG-PET/CT: ^18^F-fluoro-2-deoxy-D-glucose-positron emission tomography/computed tomography  ^6^ ERCP: endoscopic retrograde cholangiopancreatography  ^7^ HPB: hepato-pancreato-biliary system  ^8^ DSA: digital subtraction angiography | | | | |
